# Health Literacy and Fentanyl Awareness in Nursing Professionals

**DOI:** 10.1192/j.eurpsy.2026.10866

**Published:** 2026-07-31

**Authors:** F. Pastore, F. Basho, E. Domenicone, L. Domeneck, A. Litta

**Affiliations:** 1 Department of Translational Biomedicine and Neuroscience (DiBraiN), University of Bari “Aldo Moro”; 2 Department of Precision and Regenerative Medicine and Ionian Area (DiMePre-J), University of Bari “Aldo Moro”, Bari; 3 Department of Medical Specialties – Neurology Unit, San Filippo Neri Hospital, ASL Roma 1, Roma; 4 Interdisciplinary Department of Medicine, University of Bari “Aldo Moro”, Bari; 5 Mental Health Department, Asl Taranto, Taranto, Italy

## Abstract

**Introduction:**

The rising prevalence of synthetic opioids, particularly fentanyl, represents a global public health challenge. Nurses and nursing students are frontline professionals in prevention, overdose recognition and emergency management. Health Literacy (HL), the ability to access, understand and apply health information, may influence preparedness in this context.

**Objectives:**

This study aimed to assess knowledge, awareness and health literacy regarding fentanyl and drug abuse among Italian nurses and nursing students, and to examine how HL relates to preparedness in managing opioid-related risks.

**Methods:**

A multicentre cross-sectional online survey was conducted between October 2024 and January 2025 with 157 participants across Italy. The structured questionnaire included socio-demographics, the HLS19-Q12 health literacy instrument, items on drug addiction and fentanyl-specific knowledge. Descriptive and inferential statistics (Mann–Whitney U, Kruskal–Wallis H, Spearman’s rho) were applied.

**Results:**

Participants showed good baseline knowledge of fentanyl clinical use and adverse effects, but misconceptions persisted—particularly about lethal dosages and the ability to detect fentanyl through the senses. Awareness of naloxone was high (89.8%), although some participants incorrectly assumed its administration replaced the need for emergency services. HL scores did not consistently predict higher knowledge: in some cases, participants with lower HL outperformed those with higher HL on specific items such as routes of administration and naloxone use. A significant negative correlation was observed between HL and knowledge of drug availability (r = –0.35; p < 0.001). No significant differences emerged between students and professionals.

**Conclusions:**

Findings suggest that general HL may not adequately capture the contextual and experiential competencies needed for opioid-related emergencies. Nursing curricula should integrate practical, simulation-based training on fentanyl and overdose response, reframing HL as an operational clinical competence. Further studies with larger and more diverse samples are needed.

**Disclosure of Interest:**

None Declared

